# Effect of Early Post-Transplantation Tacrolimus Concentration on the Risk of Acute Graft-Versus-Host Disease in Allogenic Stem Cell Transplantation

**DOI:** 10.3390/cancers13040613

**Published:** 2021-02-04

**Authors:** Nidhi Sharma, Qiuhong Zhao, Bin Ni, Patrick Elder, Marcin Puto, Don M. Benson, Ashley Rosko, Maria Chaudhry, Srinivas Devarakonda, Naresh Bumma, Abdullah Khan, Sumithira Vasu, Samantha Jaglowski, Basem M. William, Alice Mims, Hannah Choe, Karilyn Larkin, Jonathan Brammer, Sarah Wall, Nicole Grieselhuber, Ayman Saad, Sam Penza, Yvonne Efebera

**Affiliations:** 1 Comprehensive Cancer Center Columbus, Division of Hematology, Department of Internal Medicine, The Ohio State University, Columbus, OH 43210, USA; qiuhong.zhao@osumc.edu (Q.Z.); bin.ni@vumc.org (B.N.); patrick.elder@osumc.edu (P.E.); Don.benson@osumc.edu (D.M.B.); Ashley.Rosko@osumc.edu (A.R.); maria.chaudhry@osumc.edu (M.C.); srinivas.devarakonda@osumc.edu (S.D.); naresh.bumma@osumc.edu (N.B.); Abdullah.Khan@osumc.edu (A.K.); Sumithira.Vasu@osumc.edu (S.V.); samantha.jaglowski@osumc.edu (S.J.); basem.william@osumc.edu (B.M.W.); alice.mims@osumc.edu (A.M.); hannah.choe@osumc.edu (H.C.); karilyn.larkin@osumc.edu (K.L.); jonathan.brammer@osumc.edu (J.B.); sarah.wall@osumc.edu (S.W.); nicole.Grieselhuber@osumc.edu (N.G.); Ayman.saad@osumc.edu (A.S.); sam.penza@osumc.edu (S.P.); Yvonne.Efebera@osumc.edu (Y.E.); 2 Department of Pharmacy, The Ohio State University, Columbus, OH 43210, USA; marcin.puto@osumc.edu

**Keywords:** tacrolimus, graft versus host disease, allogeneic stem cell transplantation, relapse

## Abstract

**Simple Summary:**

Allogeneic hematopoietic stem cell transplantation is a potentially curative treatment for many hematological malignancies and disorders but is often complicated by a relapse of the underlying disease, graft-vs-host disease and infectious complications. However, despite the introduction of calcineurin inhibitors such as tacrolimus, graft-versus-host disease remains one of the major life-threatening complications of allogeneic hematopoietic stem cell transplantation. Due to a variety of factors, there is variability in tacrolimus concentrations during the early weeks post-transplantation. Since the immunologic events leading to acute GVHD also occur in the first few days post-transplantation, it is important that optimal levels be attained early after transplantation. The findings from this study will help inform the management of optimal tacrolimus levels to be attained early post-transplantation.

**Abstract:**

Acute graft versus host disease (aGVHD) remains a leading cause of morbidity and mortality in allogeneic hematopoietic stem cell transplant (allo-HSCT). Tacrolimus (TAC), a calcineurin inhibitor that prevents T-cell activation, is commonly used as a GVHD prophylaxis. However, there is variability in the serum concentrations of TAC, and little is known on the impact of early TAC levels on aGVHD. We retrospectively analyzed 673 consecutive patients undergoing allo-HSCT at the Ohio State University between 2002 and 2016. Week 1 TAC was associated with a lower risk of aGVHD II–IV at TAC level ≥10.15 ng/mL (*p* = 0.03) compared to the lowest quartile. The cumulative incidence of relapse at 1, 3 and 5 years was 33%, 38% and 41%, respectively. TAC levels at week 2, ≥11.55 ng/mL, were associated with an increased risk of relapse (*p* = 0.01) compared to the lowest quartile. Subset analysis with acute myeloid leukemia and myelodysplastic syndrome patients showed significantly reduced aGVHD with TAC level ≥10.15 ng/mL at week 1 and a higher risk of relapse associated with week 2 TAC level ≥11.55 ng/mL (*p* = 0.02). Hence, achieving ≥10 ng/mL during the first week of HCT may mitigate the risk of aGVHD. However, levels (>11 ng/mL) beyond the first week may be associated with suppressed graft versus tumor effect and higher relapse.

## 1. Introduction

Acute graft versus host disease (aGVHD) remains a leading cause of morbidity and mortality in allogeneic hematopoietic stem marrow transplant (allo-HSCT), with rates ranging from 30% to 70% [1,2]. Preventing GVHD without impairing the graft-versus-tumor effect remains an important goal for successful allo-HSCT. Tacrolimus (TAC), a calcineurin inhibitor that prevents T-cell activation, is commonly used for aGVHD prophylaxis. It has been shown to be effective against T cells and has been used for GVHD prophylaxis mostly in combination with methotrexate [3,4,5,6,7].

While the influence of TAC has proved effective for preventing aGVHD after allo-HSCT, TAC varies by whole blood level, which may affect its activity/benefit [8]. The target ranges of the blood concentration of TAC early after transplantation have varied significantly among studies [5,9]. Adjusting dose levels is important to achieve therapeutic concentrations and to reduce higher blood levels that can be associated with toxicity [8,10]. Przepiorka et al. reported that mean TAC level >20 ng/mL is associated with increased risk of creatinine >2 mg/dL [10]. A pediatric study showed that a TAC concentration of ≤7 ng/mL was associated with aGVHD [11]. A study in the adult population reported that the early post-transplantation blood concentrations of TAC had a significant impact on the development of aGVHD [12], with another reporting that a level of <5 ng/mL was associated with increased aGVHD [13]. In an attempt to understand the impact of early (first four weeks) TAC levels on aGVHD incidence, Ganetsky et al. showed in 120 patients undergoing allo HSCT that had a TAC concentration of ≥12 ng/mL during the first week post-transplantation was associated with reduced risk of aGVHD [14]. However, Mori et al. showed that in 60 patients undergoing allo-HSCT, the mean blood concentration of TAC (17.3 ± 2.1) during the third week after allo-HSCT was significantly associated with lower aGVHD [12]. In addition, one of the risk factors for the development of GVHD after allo-HSCT is the use of peripheral blood (PB) cells as a graft source. Along with cyclosporine inhibitor, antihuman T-lymphocyte immune globulin (ATG) has been shown to lower the incidence of GVHD after allo-HSCT from unrelated donors [15,16]. Given these recent findings from small retrospective studies and the fact that the immunologic events that lead to aGVHD occur within the first few days after transplant, we sought to address whether early TAC levels in the first four weeks following allo-HSCT are associated with aGVHD in a larger cohort of patients who underwent allo-HSCT at The Ohio State University. We also characterize the association between TAC concentration and GVHD in patients with or without ATG.

Allo-HSCT plays a key role in the post-remission therapy for acute myeloid leukemia (AML) patients due to its high rates of efficacy as compared to alternate therapies. For patients with relapsed/refractory AML and those with high-risk myelodysplastic syndrome (MDS), it remains the sole curative option. However, these patients continue to have significant obstacles for a successful transplant, including risk for relapse of an underlying disease, GVHD, and infectious complications. Impact of cyclosporine A dose on outcome after MA allo-HSCT for AML [17,18,19,20] and TAC has been shown in refractory AML patients [21]. Approximately fifty percent of the patient population in our study were AML/MDS patients. Hence, we also analyzed the association of TAC and GVHD in this population.

## 2. Results

### 2.1. Patient Characteristics

The variables related to patient, disease and transplant are summarized in Table 1. Among the 673 patients, the median age was 53 years (range: 19–75), and 61.5% were male. In all, 68.5% of patients received reduced-intensity conditioning (RIC), and the remaining 31.5% received myeloablative conditioning (MA). The median age of donors was 36 years (range: 18–81), with 75% male. Of the donors, 36.9% were match related and 56.0% match unrelated. PB was the stem cell source for 92.4% of the patients. A total of 422 (62.7%) patients received ATG. Acute myeloid leukemia accounted for 37.2% of transplants, followed by non-Hodgkin lymphoma (17.1%), myelodysplastic syndrome (11.3%), and acute lymphoblastic leukemia (12.2%).

### 2.2. Acute GVHD and Chronic GVHD

**Figure 1:**
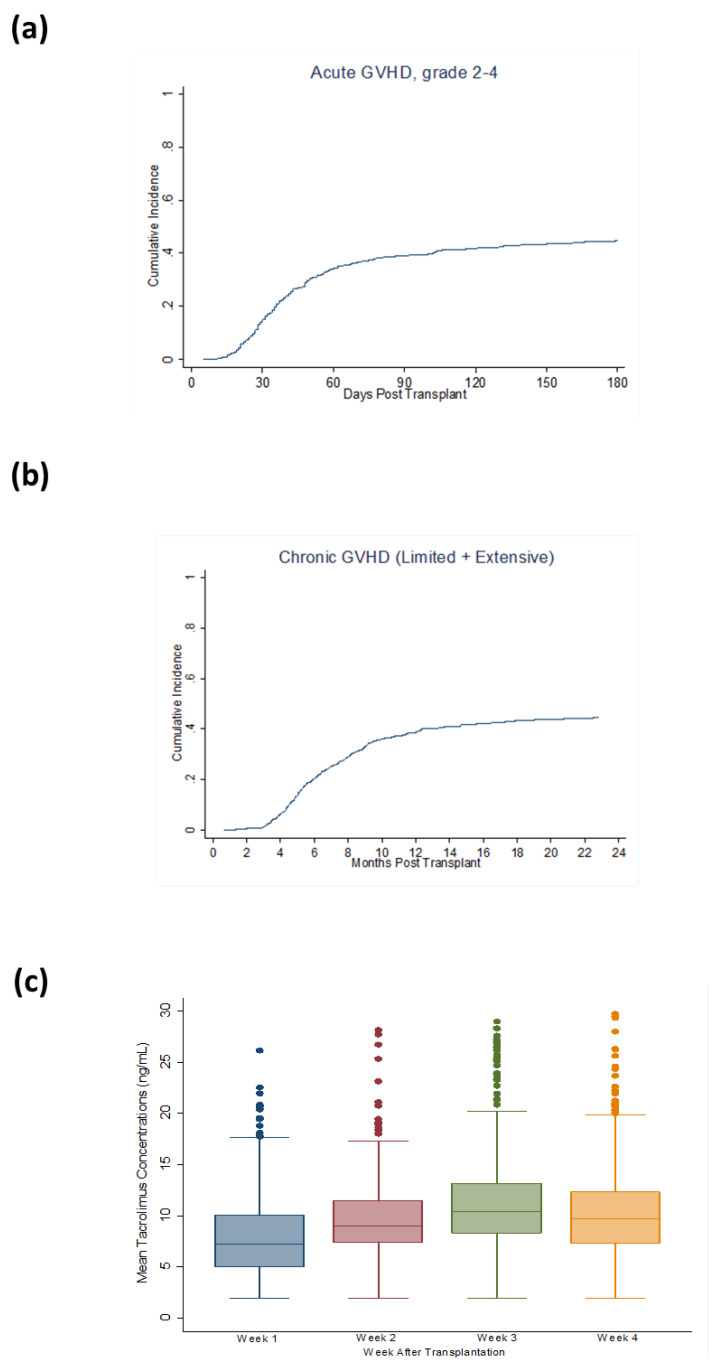
Cumulative incidence of (**a**)grade II-IV aGVHD (**b**) cGVHD and (**c**) mean weekly tacrolimus levels.

**Figure 2:**
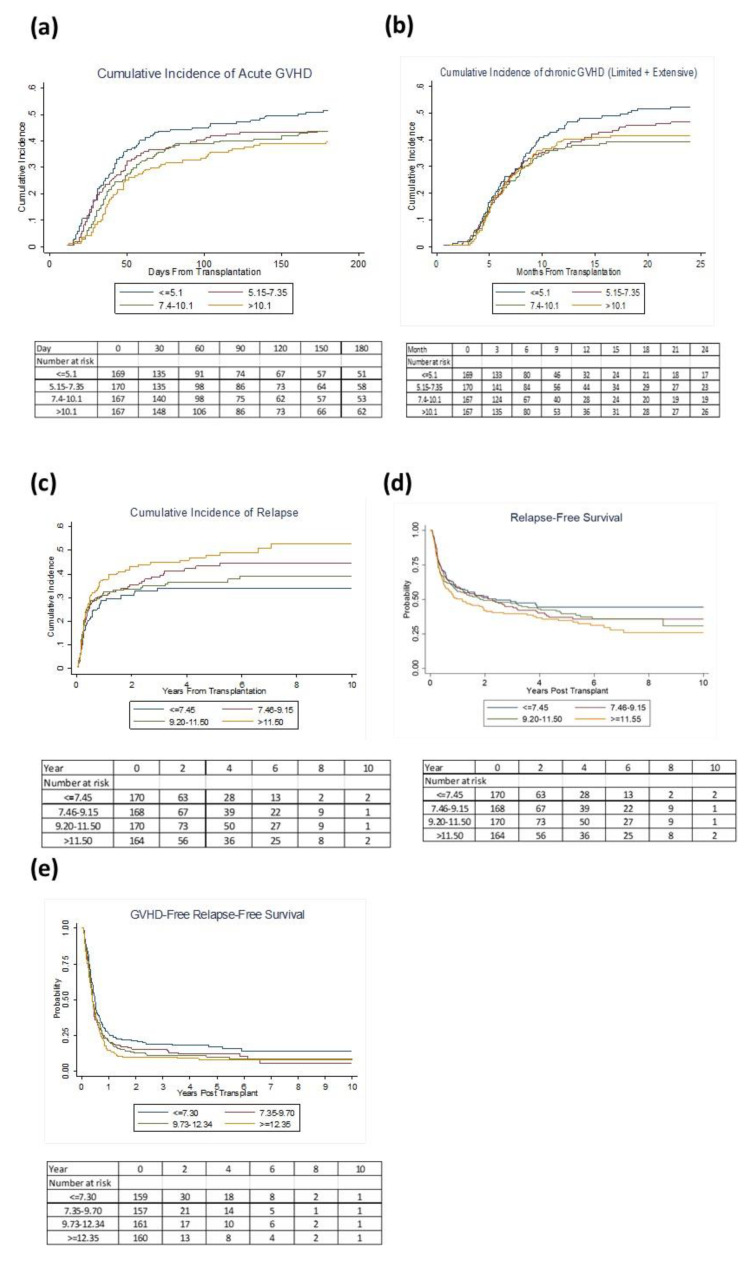
Association of TAC levels with the cumulative incidence of (**a**) grade II-IV aGVHD, (**b**) cGVHD, (**c**) relapse, and probability of (**d**) relapse free survival, (**e**) GVHD free relapse free survival.

The primary outcome of interest was aGVHD. The cumulative incidence of grades II–IV aGVHD was 40% (95% confidence interval (95% CI): 36–43%) at day 100 and 45% (95% CI: 41–48%) at day 180 post-transplant. The Cumulative incidence of Grade III–IV aGVHD was 11% (95% CI: 8–13%) at day 100 and 13% (95% CI: 11–16%) at day 180 post-transplantation. The cumulative incidence of chronic (cGVHD) was 39% (95% CI, 36–43%) at 1 year and 45% (95% CI, 41–49%) at 2 years post-transplantation (Figure 1a,b). We first analyzed the mean weekly TAC concentrations as continuous variables for the first 4 weeks post-transplantation and then determined the association of TAC levels with GVHD. The mean weekly TAC concentrations at weeks 1, 2, 3 and 4 were 8.0, 9.6, 11.1 and 10.2 ng/mL, respectively (Figure 1c). At week 1, continuous TAC concentrations were associated with lower aGVHD (H, 0.96, 95% CI 0.93–0.99, *p* = 0.016). We then examined the effect of week 1 TAC levels categorized into quartiles (<5.1, 5.15–7.35, 7.4–10.1 and >10.15 ng/mL). A higher level of TAC (≥10.15 ng/mL) was associated with a lower risk of aGVHD (Figure 2a) compared to the lowest quartile of TAC (<5.1 ng/mL). In multivariable analysis, week 1 TAC levels ≥10.15 ng/mL remained associated with a lower risk of grade II–IV aGVHD, adjusting for conditioning regimen and donor type (H, 0.70 95% CI, 0.51–0.96; *p* = 0.03; Table 2). TAC levels categorized in quartiles at week 1 also showed levels ≥10.15 ng/mL to be associated with a lower risk of aGVHD III–IV (H = 0.46 95% CI, 0.24–0.86; *p* = 0.02; Table 2).

Similarly, we determined the association of TAC levels and cGVHD. The overall rate of cGVHD at 1 and 2 years post-allo-HSCT were 39 and 45%, respectively, with that of extensive cGVHD being 36% and 42%, respectively. Week 1 TAC levels were marginally associated with a lower risk of cGVHD (H, 0.97 (95% CI: 0.94–1.01, *p* = 0.12). TAC levels categorized in quartiles at week 1 showed levels between 7.4 and 10.1 ng/mL to be marginally associated with a lower risk of cGVHD (H = 0.75 95% CI, 0.54–1.03; *p* = 0.07; Figure 2b). Furthermore, week 1 TAC levels between 7.4 and 10.1 ng/mL were marginally associated with a lower risk of extensive cGVHD (H, 0.73 95% CI, 0.52–1.01; *p* = 0.06), adjusting for age, donor sex, Karnofsky score and ATG dose.

### 2.3. Relapse and Survival

Since GVHD is linked with the graft-versus-tumor effect, we examined whether early TAC levels influenced the risk of disease relapse. The cumulative incidence of relapse at 1, 3 and 5 years post-allo-HSCT was 33% (95% CI: 29–36%), 38% (95% CI: 34–42%) and 41% (95% CI: 37–44%), respectively. TAC levels at week 1 were not associated with relapse. However, week 2 showed an incidence of disease relapse was associated with TAC levels. Multivariable analysis showed the upper Quartile (≥11.55 ng/mL) to be associated with a higher risk of relapse (H, 1.60, 95% CI, 1.12–2.29, *p* = 0.01) compared to the lowest TAC quartile, after adjusting for confounding variables (Figure 2c and Table 3). Similarly, we examined whether TAC concentrations were associated with survival. The 1, 3 and 5 years relapse-free survival was 56%, 45% and 39%, respectively. TAC levels categorized in quartiles at week 2 showed levels ≥11.55 ng/mL to be associated with a higher risk of relapse or death (H = 1.39 95% CI, 1.03–1.86; *p* = 0.03; Figure 2d, Table 3). The probabilities of GRFS at 1, 3 and 5 years were 20%, 13% and 11%, respectively. Only week 4 mean TAC level was associated with GRFS (H, 1.02, 95% CI, 1.001–1.04, *p* = 0.041). At week 4, TAC levels, ≥9.73 ng/mL were associated with a higher risk of GVHD, relapse or death (H, 1.29, 95% CI, 1.01–1.65, *p* = 0.042 for TAC levels of 9.73–12.34, and H 1.42, 95% CI, 1.11–1.82, *p* = 0.005 for TAC levels of >12.35; Figure 2e,). At a median follow of 3.8 years among the surviving patients, TAC levels at weeks 1, 2, 3 and 4 were not associated with OS.

### 2.4. Subset Analysis on AML/MDS Patients

**Figure 3:**
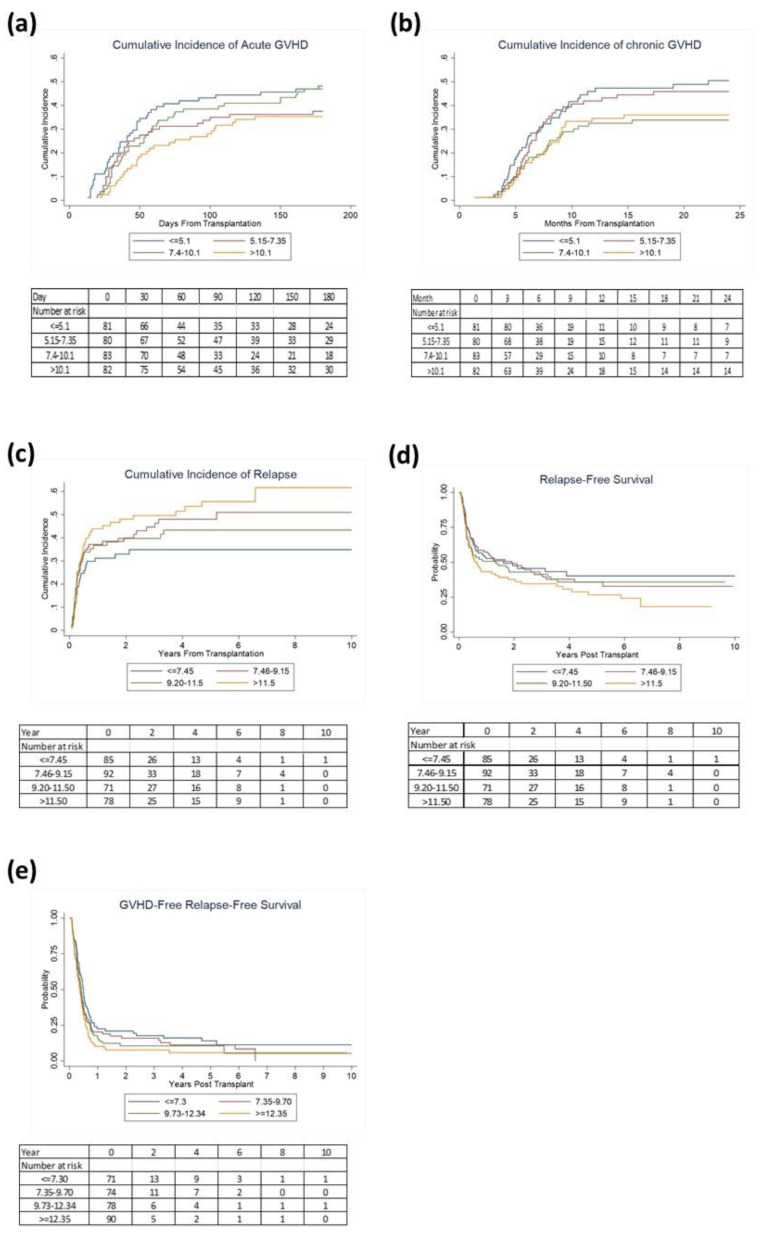
Association of TAC levels with the cumulative incidence of (**a**) grade II-IV aGVHD, (**b**) cGVHD, (**c**)relapse, and probability of (**d**) relapse free survival, (**e**) GVHD free relapse free survival in AML/MDS patients

The median age of the AML/MDS patients was 56 years (range: 19–75). Sixty-six percent of patients received reduced-intensity conditioning, and ninety percent of patients had PB as the graft source (Supplemental Table S1). At week 1 continuous TAC concentrations were associated with lower aGVHD (H, 0.96, 95% CI 0.93–0.99, *p* = 0.005). To better delineate the association between week 1 TAC levels and aGVHD, we further categorized TAC levels in quartiles. Compared to the lowest TAC quartile, TAC levels ≥ 10.15 ng/mL remained marginally associated with a lower risk of aGVHD with a slightly lower risk of grade II–IV aGVHD, after adjusting for conditioning, related donors (H, 0.64 95% CI, 0.40–1.04; *p* = 0.07; Figure 3a). TAC levels categorized in quartiles at week 1 also showed levels ≥10.15 ng/mL were associated with a lower risk of aGVHD III–IV (H = 0.30 95% CI, 0.11–0.84; *p* = 0.02). Further, multivariable analysis at week 2 showed the upper quartile (≥11.55 ng/mL) was associated with a higher risk of relapse (H, 1.81, 95% CI, 1.09–3.01, *p* = 0.02; Figure 3c) compared to the lowest quartile, after adjusting for other confounding variables (Table 4).

### 2.5. Effect of Tacrolimus Levels on Outcome in Patients with and without ATG

To further characterize the association between TAC concentration and GVHD, we analyzed patients from 2 cohorts: with ATG (*n* = 422) and no- ATG (*n* = 251). The majority of the patients in the no-ATG group had related donors (90%), and the ATG group consisted of mostly unrelated donors (93%; Supplemental Table S2). PB was the graft source for most patients in both groups (95%-no ATG) and 91%-ATG).

Among patients who received ATG, the cumulative incidence of aGVHD, II–IV was 45% and 49%, respectively, at day 100 and day 180 post-transplantation. The cumulative incidence of aGVHD III–IV was 13% and 14% at day 100 and day 180, respectively. The cumulative incidence of cGVHD (limited and extensive) at 1 and 2 years post-transplantation was 37% and 42%, respectively. The 1 and 2 year cGVHD extensive rates were 32 and 37%, respectively. Further, the relapse rates were 25%, 32% and 35% at 6 months, 1 and 2 years post-transplantation.

The week 1 TAC levels ≥10.15 ng/mL remained associated with a marginally lower risk of grade II–IV aGVHD, adjusting for conditioning, related donors (H, 0.68 95% CI, 0.46–1.01; *p* = 0.06). Further, TAC at week 1 also showed levels ≥10.15 ng/mL to be associated with a lower risk of aGVHD III–IV (H = 0.30 95% CI, 0.12–0.76; *p* = 0.01). Week 1 TAC levels were also associated with both cGVHD (limited and extensive) and cGVHD (extensive). Week 1 TAC levels of ≥10.1 ng/mL were shown to be significantly associated with lower cGVHD (extensive), H = 0.45, 95% CI, 0.28–0.74, *p* = 0.001 after adjusting for age, donor gender and Karnofsky score. Week 2 showed upper quartile (≥11.55 ng/mL) was associated with a higher risk of relapse (H, 2.15, 95% CI, 1.37–3.37, *p* < 0.01) after adjusting for other confounding variables (Table 5).

Analysis of aGVHD and relapse among patients who did not receive ATG showed no significant associations between TAC and aGVHD II–IV, aGVHD III–IV, or relapse. The fact that patients without ATG had mostly related donors, while patients receiving ATG had mostly unrelated donors, agrees with the observation that the TAC effect only retained in patients with unrelated donors.

## 3. Patients and Methods

We performed a retrospective study of 673 adult patients with hematological malignancies undergoing allo-HSCT at the Ohio State University between January 2002 and December 2016. All patients received TAC as part of GVHD prophylaxis.

### 3.1. GVHD Prophylaxis

All MA allo-HSCT patients received standard prophylaxis with TAC IV 0.02 mg/kg/day IV continuous infusion starting day–2 and methotrexate 15 mg/m^2^ on day +1 and 10 mg/m^2^ on days +3, +6, +11. Rabbit ATG was given for unrelated patients only. For RIC, allo-transplant oral TAC 0.06 mg/kg/day in two divided doses was given along with mini methotrexate 5 mg/m^2^ on days +1, +3, +6, +11 for unrelated transplants along with rabbit ATG and mini methotrexate on days +1, +3, +6 for related transplant. TAC blood levels were monitored initially twice weekly, then once weekly until day +100 to maintain blood levels between 5 and 12 ng/mL. If no aGVHD by day 100, TAC was gradually weaned off by day 180 post-transplant. Serological concentrations of TAC were measured using an automated microparticle enzyme immunoassay. Mean TAC concentrations were calculated from the first week post-transplantation to 4 weeks post-transplantation.

### 3.2. Study End Points

The primary outcome of interest was the incidence of aGVHD and its association with the mean weekly TAC levels. Secondary endpoints included incidence of chronic GVHD (cGVHD), GVHD-free, relapse-free survival (GRFS), relapse, progression-free survival (PFS), and overall survival (OS). Grading of aGVHD and cGVHD were done using the consensus conference criteria [22] and the National Institute of Health Consensus Development Project Criteria, respectively [23,24]. The mean TAC concentration for each of the first four weeks post-allo-HSCT was used. Cumulative incidence rates of aGVHD were estimated within the first 180 days post-transplant. Relapse was defined as the time from transplant to relapse, and patients were censored at the last clinical assessment if no relapse. Progression-free survival (PFS) was defined as the time from transplant until relapse or death, whichever occurred first. GRFS was defined as the time from transplant until relapse, grade II to IV aGVHD, cGVHD or death, whichever occurred first. Overall, survival (OS) was defined as the time from transplant until death. Competing risks for aGVHD and cGVHD were relapse or death, while the competing risk for relapse is an early death.

### 3.3. Statistical Analysis

Fine and Gray’s proportional hazard models accounting for competing risks were used to evaluate the association between TAC levels and outcome of aGVHD, cGVHD and relapse. Cox proportional hazard models were used for the association with PFS, GRFS and OS. Mean weekly TAC levels were included in the analyses as continuous variables and then divided into Quartiles. The following were evaluated as potential confounding factors: age, sex, gender, disease type, donor source, degree of HLA match, remission status, Karnofsky score, and conditioning regimen. Univariable models were conducted first to evaluate the associations between each potential confounding factor and clinical outcome. The factors with univariable *p*-values less than 0.20 were further evaluated in a multivariable analysis using a stepwise method retaining only statistically significant variables in the final model. The significance was set at *p* < 0.05.

## 4. Discussion

This is the largest study to date analyzing the association between TAC concentrations and clinical outcomes, including aGVHD from 673 patients given allogeneic hematopoietic cell grafts. To minimize the risk of GVHD, calcineurin inhibitors are typically targeted to higher levels. Our results showed variability in TAC levels in the first four weeks post-transplantation and that week 1 TAC levels were associated with aGVHD and cGVHD. Specifically, TAC levels of ≥10.15 ng/mL were associated with a lower risk of grade II–IV and III–IV aGVHD. In contrast to the findings of others [14,25,26], we did observe TAC levels ≥10.15 ng/mL were also marginally associated with a lower incidence of extensive cGVHD. Non-myeloablative conditioning appears to have minimum tissue injury [27], a transient state of mixed chimerism [28], a different prophylaxis regimen [29], which could, in turn, lead to delayed GVHD. In addition, a non-ablative regimen has been indicated to have a higher number of antigen-presenting cells and thus may play a role in early GVHD response post-transplantation [30,31]. Given the possibility of minimum tissue injury after reduced-intensity, it is a possibility that higher TAC levels may have had a therapeutic effect in our cohort. These results remained true when we analyzed patients with AML/MDS only, the largest group getting allo-HSCT with a standard conditioning regimen. On further analysis, TAC levels early post-transplant have no effect in related donor transplants. In a 2005 preclinical study, Beilhack et al. [32] have shown that initiating events of acute GVHD occur early post-transplantation. These results further show the importance of achieving target TAC concentrations early after allotransplantation.

Freedom from ongoing morbidity is an important outcome for post-HSCT. Relapse and GRFS are meaningful endpoints for evaluating GVHD prophylaxis after HSCT. The results from our study showed that week 2 TAC levels were associated with relapse or death. No association between TAC levels was observed with GRFS at week 1. However, higher week 4 TAC levels (≥9.74 ng/mL) were associated with a higher risk of GVHD, relapse or death. Overall, we found that TAC level ≥10 during the first week of HCT may mitigate the risk of aGVHD. However, higher levels (>11) beyond the first week may be associated with suppressed graft versus tumor effect and higher relapse. Thus, the timing of administration of GVHD prophylaxis drugs appears to be important in controlling GVHD.

Our cohort, though it was uniform in the GVHD prophylaxis given and graft source, had the limitations like any other retrospective study of a heterogeneous population: with different routes of TAC administration and heterogeneity in the conditioning regimen. Similar results were reported by Ganetsky et al., where they showed that TAC concentrations >12 ng/mL during the first week after allotransplantation was associated with significantly reduced risk of II–IV aGVHD without increasing risk of relapse. In contrast to our study, Ganetsky et al. [14] did not find any association between TAC and cGVHD or relapse. Another difference was that all patients received reduced-intensity conditioning, whereas, in our study, there was a mix of patients receiving myeloablative or reduced-intensity conditioning. Another retrospective study reported no correlation between TAC concentrations and acute grade II–IV/III–IV GVHD at week 1 with myeloablative conditioning but found levels <10.5 ng/mL to be associated with inferior control of III–IV aGVHD with non-myeloablative conditioning [25]. In addition, no correlation between week 2 means TAC concentrations and risk of relapse was determined with both myeloablative and non-myeloablative regimens [25].TAC is metabolized by enzymes that are polymorphic and have gene variants that influence how it could be metabolized at different rates in the population [33]. In addition, depending upon the circumstances, the route of administration can be difficult to keep consistent between patients. Hence, one potential approach could be to map the genotypes to TAC metabolism rates before initiating TAC.

In summary, our study is the largest one assessing the importance of TAC concentrations within the first four weeks after allogeneic HSCT and GVHD outcomes. Based on our observations, patients undergoing allogeneic transplantation should be targeted at a TAC level of 10.15–11.55 ng/mL within the first four weeks of transplant to reduce the risk of acute and cGVHD without increasing relapse rate. The utility of optimizing TAC concentrations early after transplantation should be validated in prospective studies.

## 5. Conclusions

Acute GVHD remains a leading cause of morbidity and mortality in allo-HSCT. Tacrolimus, a calcineurin inhibitor that prevents T-cell activation, is commonly used as a GVHD prophylaxis; however, there is variability in the serum concentrations of TAC, and little is known on the impact of early TAC levels on aGVHD. Our study is the largest to date, analyzing the association between TAC concentrations and clinical outcomes from 673 patients undergoing allo-HSCT. Our results showed the following: 1: that TAC levels of ≥10.15 ng/mL during the first week of allo-SCT was associated with a lower risk of grade II–IV and III-1 V aGVHD; 2: Higher levels of TAC (>11 ng/mL) beyond the first week may be associated with suppressed graft versus tumor effect and higher relapse; and 3: This association between TAC levels and GVHD was retained with unrelated donors. The utility of optimizing TAC concentrations early after transplantation should be validated in prospective studies.

## Figures and Tables

**Table 1:** Patient and donor characteristics.

**Patient Characteristics (*n* = 673)**	**N**	**%**
Age, patient, median, range	53	19–75
Age, donor, median, range	36	18–81
Gender, patient		
Female	259	38.48
Male	414	61.52
Gender, donor		
Female	168	24.96
Male	505	75.04
Ethnic group		
African American	27	4.01
Caucasian	641	95.25
Others	5	0.74
Conditioning		
MA	212	31.5
RIC	461	68.5
Tissue		
BM	51	7.58
PB	622	92.42
Disease		
Acute lymphoblastic leukemia	82	12.18
Acute myeloid leukemia	250	37.15
Chronic lymphocytic leukemia	41	6.09
Chronic myeloid leukemia/chronic myelomonocytic leukemia	30	4.46
Hodgkin’s disease	25	3.71
Multiple myeloma	16	2.38
Non-Hodgkin’s lymphoma	115	17.09
Myelodysplastic syndrome	76	11.29
BPDC/MF/PLL/MPD	38	5.65
Remission status		
Complete response	334	49.63
Partial response	131	19.47
Primary refractory	40	5.94
Persistent disease	128	19.02
Relapse	40	5.94
Response post SCT		
Complete response	517	76.82
Partial response/very good partial response	11	1.63
Persistent disease	91	13.25
Stable disease	41	6.09
Not evaluable	13	1.93
Karnofsky score		
60	2	0.3
70	20	2.97
80	186	27.64
90	351	52.15
100	114	16.94
Comorbidity index (category)		
0–1	218	33.96
2–3	247	38.47
4–5	131	20.4
>5	46	7.17
missing	31	
ATG dose		
3	5	0.74
4	22	3.27
4.5	125	18.57
6	221	32.84
7.5	49	7.28
no ATG	251	37.3
GVHD prophylaxis		
FKMTX	673	100
Donor		
Match related	248	36.85
Match unrelated	377	56.02
Mismatch related	6	0.89
Mismatch unrelated	42	6.24
CD34, median, range	6.6	0.7–93.1
Cd3, median, range	2.4	0–9.5

Abbreviations: MA, myeloablative; RIC, reduced-intensity conditioning; BM, bone marrow; PB, peripheral blood; BPDC/MF/PLL/MPD, blastic plasmacytoid dendritic cell neoplasia/myelofibrosis/polymorphocytic leukemia/myeloproliferative disorder; ATG, anti-thymocyte globulin.

**Table 2:** Multivariable analysis for factors affecting the incidence of aGVHD and cGVHD.

**Association with aGVHD II–IV**	HR	95% CI	*p*	
MVA				
Week 1 tacrolimus quartiles				
≤5.1	Reference			
5.15–7.35	0.82	0.60	1.12	0.20
7.4–10.1	0.81	0.59	1.10	0.17
10.15–26.2	0.70	0.51	0.96	0.03
Unrelated vs. related	1.76	1.38	2.25	<0.01
RIC vs. MA	0.73	0.58	0.92	0.01
**Association with aGVHD III–IV**				
MVA				
Week 1 tacrolimus quartiles				
≤5.1	Reference			
5.15–7.35	0.60	0.34	1.06	0.08
7.4–10.1	0.90	0.53	1.52	0.68
10.15–26.2	0.46	0.24	0.86	0.02
Unrelated vs. related	1.85	1.16	2.96	0.01
**Association with Chronic GVHD, Extensive**				
MVA				
Week 1 tacrolimus quartiles				
≤5.1	Reference			
5.15–7.35	0.87	0.63	1.20	0.38
7.4–10.1	0.73	0.52	1.01	0.06
10.15–26.2	0.73	0.52	1.03	0.07
Age	0.99	0.98	1.00	0.07
Donor sex: male vs. female	0.66	0.51	0.86	0.002
Karnofsky score	1.02	1.01	1.04	0.002
ATG dose				
No ATG	0.90	0.65	1.26	0.54
3 or 4	1.12	0.66	1.90	0.69
4.5	Reference			
6	0.64	0.45	0.92	0.02
7.5	0.40	0.21	0.77	0.01

Abbreviations: MVA, multivariable analysis; aGVHD, acute graft versus host disease; cGVHD, chronic graft versus host disease; RIC, reduced-intensity conditioning; MA, myeloablative; ATG, Anti-thymocyte globulin.

**Table 3:** Multivariable analysis for factors affecting the incidence of relapse.

**Modeling on Relapse**	HR	95% CI		*p*
MVA				
Week 2 tacrolimus quartiles				
≤7.45			Reference	
7.46–9.15	1.34	0.93	1.93	0.12
9.20–11.50	1.20	0.82	1.76	0.35
11.55–28.15	1.60	1.12	2.29	0.01
Donor sex: Male vs. Female	1.43	1.05	1.94	0.02
RIC vs. MA	1.38	1.00	1.89	0.05
Disease				
ALL			Reference	
AML/MDS	1.71	1.07	2.73	0.03
CLL	0.79	0.36	1.74	0.56
HL/NHL	0.75	0.39	1.43	0.38
MPD and others	1.40	0.76	2.58	0.28
Remission status				
Complete response			Reference	
Partial response	1.99	1.22	3.23	0.01
Primary refractory	2.08	1.30	3.34	0.002
Persistent disease	0.94	0.64	1.38	0.75
Relapse	2.80	1.72	4.56	<0.001
Karnofsky Score	0.98	0.96	1.00	0.02

Abbreviations: MVA, multivariable analysis; RIC, reduced-intensity conditioning; MA, myeloablative; ALL, Acute lymphoblastic leukemia; AML/MDS, acute myeloid leukemia/myelodysplastic syndrome; CLL, Chronic lymphocytic leukemia; HL/NHL, Hodgkin’s lymphoma/non-Hodgkin’s lymphoma; MPD, myeloproliferative disorder.

**Table 4:** Multivariable analysis for factors affecting the incidence of aGVHD and relapse in AML/MDS population.

**Association with aGVHD II–IV**	HR	95% CI		*p*
MVA				
Week 1 tacrolimus quartiles				
≤5.1			Reference	
5.15–7.35	0.69	0.42	1.13	0.14
7.4–10.1	0.90	0.58	1.39	0.64
10.15–26.2	0.64	0.40	1.04	0.07
Unrelated vs. related	1.54	1.07	2.20	0.02
RIC vs. MA	0.73	0.52	1.03	0.07
**Association with aGVHD III–IV**				
MV	HR	95% CI		*p*
Week 1 tacrolimus quartiles				
≤5.1	Reference			
5.15–7.35	0.43	0.18	1.05	0.07
7.4–10.1	1.08	0.54	2.18	0.83
10.15–26.2	0.30	0.11	0.84	0.02
Unrelated vs. related	1.92	0.97	3.79	0.06
**Modeling on Relapse**				
MVA	HR	95% CI		*p*
Week 2 tacrolimus quartiles				
≤7.45			Reference	
7.46–9.15	1.57	0.95	2.60	0.08
9.20–11.50	1.49	0.86	2.59	0.16
11.55–28.15	1.81	1.09	3.01	0.02
Donor sex: Male vs. Female	1.35	0.88	2.09	0.17
RIC vs. MA	1.46	0.98	2.17	0.06
Remission status				
Complete response			Reference	
Primary refractory	1.81	1.02	3.23	0.044
Persistent disease	0.98	0.63	1.52	0.93
Relapse	2.84	1.55	5.22	0.001
Karnofsky Score	0.97	0.95	0.99	0.00

Abbreviations: MVA, multivariable analysis; aGVHD, acute graft versus host disease; RIC, reduced-intensity conditioning; MA, myeloablative; RIC, reduced-intensity conditioning; MA, myeloablative.

**Table 5:** Multivariable analysis for factors affecting the incidence of aGVHD and relapse in patients who received ATG.

**Modeling on aGVHD III–IV**	HR	95% CI		*p*
MVA				
Week 1 tacrolimus quartiles				
≤5.1			Reference	
5.15–7.35	0.59	0.30	1.16	0.13
7.4–10.1	0.98	0.53	1.84	0.96
10.15–26.2	0.30	0.12	0.76	0.01
Unrelated vs. related	4.67	0.68	32.08	0.12
**Modeling on Relapse**				
MVA	HR	95% CI		*p*
Week 2 tacrolimus quartiles				
≤7.45			Reference	
7.46–9.15	1.66	1.04	2.65	0.04
9.20–11.50	1.09	0.66	1.83	0.73
11.55–28.15	2.15	1.37	3.37	<0.01
Donor sex: Male vs. Female	1.16	0.77	1.73	0.49
RIC vs. MA	1.06	0.72	1.55	0.78
Remission status				
Complete response			Reference	
Partial response	1.62	1.04	2.52	0.03
Primary refractory	2.78	1.54	5.03	0.001
Persistent disease	0.94	0.60	1.49	0.80
Relapse	1.81	0.97	3.37	0.060
Karnofsky Score	0.97	0.94	0.99	0.01

Abbreviations: MVA, multivariable analysis; aGVHD, acute graft versus host disease; RIC, reduced-intensity conditioning; MA, myeloablative; RIC, reduced-intensity conditioning; MA, myeloablative.

## Data Availability

This is Institutional data and raw data is not publicly available.

## Supplementary Materials

The following are available online at https://www.mdpi.com/2072-6694/13/4/613/s1, Table S1: Patient Characteristics Among AML and MDS Patients, Table S2: Patient Characteristics of Patients with and without ATG.
