# Supplementary material for: Effect of Early Post-Transplantation Tacrolimus Concentration on the Risk of Acute Graft-Versus-Host Disease in Allogenic Stem Cell Transplantation
Source: Cancers (Basel). 2021 Feb 4;13(4):613. doi: 10.3390/cancers13040613 (PMC7913846; doi:10.3390/cancers13040613)
Supplement: Supplementary file 1 [file cancers-13-00613-s001.pdf]

Table S1. Patient Characteristics Among AML and MDS Patients (n = 326).

Patient Characteristics	N	%
Age, patient, median, range	56	19–75
Age, donor, median, range	34	18–81
Gender, patient		
Female	140	42.9
Male	186	57.1
Gender, donor		
Female	78	23.9
Male	248	76.1
Race		
African American	14	4.3
Caucasian	309	94.8
Others	3	0.9
Conditioning		
MA	112	34.4
RIC	214	65.6
Tissue		
BM	32	9.8
PB	294	90.2
Remission Status		
CR	213	65.3
PRIMARY REF	21	6.4
Persistent disease	70	21.5
Relapse	22	6.8
Response post SCT		
CR	280	85.9
PD	33	10.1
STABLE	9	2.8
NE	4	1.2
Karnofsky score		
60	1	0.3
70	12	3.7
80	93	28.5
90	166	50.9
100	54	16.6
Comorbidity Index (Category)		
0–1	103	32.5
2–3	113	35.6
4–5	72	22.7
>5	29	9.1
ATG dose		
**no ATG**	111	34.0
3	22	6.7
4.5	60	18.4
6	110	33.7

7.5	23	7.1
Donor		
match related	108	33.1
match unrelated	188	57.7
mismatch related	4	1.2
mismatch unrelated	26	8.0
CD34	6.4	1.6–12.0
Cd3	2.4	0–9.5

Table S2. Patient characteristics of patients with and without ATG.

**Patient Characteristics**	no ATG (*n* = 251)		ATG (*n* = 422)		*p*-value
Age, patient, median, range	52	19–75	55	19–74	0.02
Age, donor, median, range	51	18–76	29	18–81	<0.001
Gender, patient					0.28
Female	90	35.9	169	40.1	
Male	161	64.1	253	59.9	
Gender, donor					<0.001
Female	94	37.45	74	17.54	
Male	157	62.55	348	82.46	
Race					0.02
African American	15	5.98	12	2.84	
Caucasian	232	92.43	409	96.92	
Others	4	1.59	1	0.24	
Donor					<0.001
RELATED	225	89.64	29	6.87	
UNRELATED	26	10.36	393	93.13	
Conditioning					0.017
MA	93	37.05	119	28.2	
RIC	158	62.95	303	71.8	
Tissue					0.03
BM	12	4.8	39	9.2	
PB	239	95.2	383	90.8	
Disease					0.02
ALL	33	13.15	49	11.61	
AML/MDS	111	44.22	215	50.95	
CLL	8	3.19	33	7.82	
HL/NHL	65	25.9	75	17.77	
MPD and others	34	13.55	50	11.85	
Remission Status					0.12
CR	117	46.61	217	51.42	
PR	57	22.71	74	17.54	
PRIMARY REF	20	7.97	20	4.74	
Persistent disease	41	16.33	87	20.62	
Relapse	16	6.37	24	5.69	
Response post SCT					0.83
CR	192	76.5	325	77	
PR, VGPR	5	2	6	1.4	

PD	35	14	56	13.2	
STABLE	16	6.4	25	5.9	
NE	3	1.2	10	2.4	
Karnofsky score					0.27
60	2	0.8	0	0	
70	5	1.99	15	3.55	
80	68	27.09	118	27.96	
90	130	51.79	221	52.37	
100	46	18.33	68	16.11	
Comorbidity Index (Category)					0.5
0–1	78	33.62	140	34.15	
2–3	93	40.09	154	37.56	
4–5	49	21.12	82	20	
>5	12	5.17	34	8.29	
Mismatch					<0.001
Match	249	99.2	376	89.1	
Mismatch	2	0.8	46	10.9	
CD34	6	1.4–93.1	7	0.7–12.0	0.02
Cd3	2.7	0–9.5	2.2	0–5.5	<0.001
